# Susceptibility of Human Melanoma Cells to Autologous Natural Killer (NK) Cell Killing: HLA-Related Effector Mechanisms and Role of *Unlicensed* NK Cells

**DOI:** 10.1371/journal.pone.0008132

**Published:** 2009-12-04

**Authors:** Paolo Carrega, Gaetana Pezzino, Paola Queirolo, Irene Bonaccorsi, Michela Falco, Giuseppe Vita, Daniela Pende, Aldo Misefari, Alessandro Moretta, Maria Cristina Mingari, Lorenzo Moretta, Guido Ferlazzo

**Affiliations:** 1 Istituto Giannina Gaslini, Genoa, Italy; 2 Laboratory of Immunology and Biotherapy, Department of Human Pathology, University of Messina, Messina, Italy; 3 Istituto Nazionale per la Ricerca sul Cancro, Genoa, Italy; 4 Unit of Cell Typing, Department of Pathology and Experimental Microbiology, University of Messina, Messina, Italy; 5 Department of Experimental Medicine, University of Genoa, Genoa, Italy; 6 Centro di Eccellenza per le Ricerche Biomediche, University of Genoa, Genoa, Italy; Centre de Recherche Public de la Santé (CRP-Santé), Luxembourg

## Abstract

**Background:**

Despite Natural Killer (NK) cells were originally defined as effectors of spontaneous cytotoxicity against tumors, extremely limited information is so far available in humans on their capability of killing cancer cells in an autologous setting.

**Methodology/Principal Findings:**

We have established a series of primary melanoma cell lines from surgically resected specimens and here showed that human melanoma cells were highly susceptible to lysis by activated autologous NK cells. A variety of NK cell activating receptors were involved in killing: particularly, DNAM-1 and NKp46 were the most frequently involved. Since self HLA class I molecules normally play a protective role from NK cell-mediated attack, we analyzed HLA class I expression on melanomas in comparison to autologous lymphocytes. We found that melanoma cells presented specific allelic losses in 50% of the patients analyzed. In addition, CD107a degranulation assays applied to NK cells expressing a single inhibitory receptor, revealed that, even when expressed, specific HLA class I molecules are present on melanoma cell surface in amount often insufficient to inhibit NK cell cytotoxicity. Remarkably, upon activation, also the so called “unlicensed” NK cells, i.e. NK cells not expressing inhibitory receptor specific for self HLA class I molecules, acquired the capability of efficiently killing autologous melanoma cells, thus additionally contributing to the lysis by a mechanism independent of HLA class I expression on melanoma cells.

**Conclusions/Significance:**

We have investigated in details the mechanisms controlling the recognition and lysis of melanoma cells by autologous NK cells. In these autologous settings, we demonstrated an efficient *in vitro* killing upon NK cell activation by mechanisms that may be related or not to abnormalities of HLA class I expression on melanoma cells. These findings should be taken into account in the design of novel immunotherapy approaches against melanoma.

## Introduction

NK cells were initially defined on a functional basis to identify lymphoid cells capable of lysing various tumour cell lines in the absence of previous stimulation [1]. NK cell cytotoxic function is regulated by numerous activating and inhibitory receptors [2] and all available data are compatible with the concept that the ligands for activating NK receptors are expressed primarily by “stressed” cells (e.g. tumour or virus-infected cells). NKp46, NKp30 and NKp44 are activating receptors that have been collectively named “natural cytotoxicity receptors” (NCR). They represent the first activating receptors mediating NK cytotoxicity to be identified and molecularly characterized [3]. To date, limited information is available on the cell surface ligands recognized by NCR with the remarkable exception of B7 H6 ligand recognized by NKp30 [4]. A direct correlation has been established between the surface density of NCR on NK cells and the intensity of NK-mediated cytolytic activity [5]. Another activating receptor, NKG2D, is expressed not only by NK cells but also by cytotoxic T lymphocytes. NKG2D recognizes the stress-inducible MICA/B [6] and ULBPs proteins [7]. More recently, it has been shown that DNAM-1, a triggering receptor expressed by virtually all NK cells (that is partially expressed also by T lymphocytes and monocytes), specifically recognizes PVR (CD155) and Nectin-2 (CD112) [8], two members of the nectin family.

With respect to the inhibitory receptors, human NK cells express different HLA-class I-specific inhibitory receptors [2], [9]–[11]. Important receptors are represented by killer Ig-like receptors (KIR) which detect allelic determinants on HLA class I molecules. In particular, HLA-C allotypes have either the C1 epitope (shared by HLA-Cw alleles characterized by Ser77Asn80, including HLA-Cw1, -Cw3, -Cw7, -Cw8, -Cw12, -Cw13, -Cw14 and -Cw16 alleles), recognized by KIR2DL2/3, or the C2 epitope (Asn77Lys80 shared by HLACw2, -Cw4, -Cw5, -Cw6, -Cw15, Cw*-1602, -Cw17 and -Cw18), the ligand for KIR2DL1. Similarly, all HLA-B allotypes have either the Bw4 or Bw6 epitope, but only the Bw4 epitope is a ligand for KIR, its cognate inhibitory receptor being KIR3DL1 [2], [9]. Another relevant receptor, CD94/NKG2A, recognizes the nonclassical MHC class I molecule HLA-E [11]. Accordingly, NK cells lyse target cells that have lost (or express low amounts of) MHC class I molecules. This event occurs frequently in tumours or in cells infected by some viruses such as certain Herpes viruses or Adenoviruses [1], [2], [9]. Studies dealing with human NK cell cytotoxicity have been almost exclusively performed employing, as target cells, allogeneic tumour cell lines that express unrelated HLA class I repertoires. Under these experimental conditions it is not possible to assess whether changes in HLA class I expression in cancer cells may indeed affect NK cell-mediated killing (due to the possible existence of KIR-HLA class I mismatches).

Abnormalities in HLA antigen expression in malignant cells represent a relatively frequent event in cancer biology. However, most of the studies investigating HLA expression in cancer tissues were performed by immunohistochemistry, a technical approach that does not allow detection of specific HLA alleles alterations. In addition, the use of available tumor cell lines as target cells does not allow a comparative analysis of normal cells isolated from the same donors. As a consequence, it is not possible to evaluate HLA allelic losses occurred during cancer transformation. In general, it is assumed that abnormalities in HLA expression are extremely variable in human cancers ranging from 0 to 90% [12]. They are caused by distinct mechanisms, which include defects in β_2_-microglobulin synthesis, loss of the gene(s) encoding HLA antigen heavy chain(s), mutations which inhibit the HLA-class I transcription or translation, defects in the regulatory mechanisms controlling HLA antigen expression and/or abnormalities in antigen processing [12]–[14].

It has long been believed that all NK cells express at least one receptor with specificity for self-MHC class I [10], [15]. This concept was originally based on studies performed primarly on NK cell clones and on a limited number of donors. More recently, studies from different laboratories have shown that some NK cells may not express any self-MHC class I-specific inhibitory receptor [16], [17]. These include both NK cells expressing no inhibitory KIR or NKG2A and NK cells that only express inhibitory KIR not recognizing self HLA class I molecules. In humans, a substantial fraction of normal peripheral blood (PB) NK cells (∼13%) lack both self HLA class-I specific receptors KIR and NKG2A [17]. Remarkably, these NK cells (named hereafter “HLA non-inhibited NK”) display low cytolytic activity even against the HLA class I-deficient target cells K562 [17] that are highly susceptible to NK cell-mediated lysis. However, the role and the activity of “HLA non-inhibited” (the so called “unlicensed”) NK cells has never been tested against autologous target cells expressing HLA class I.

To date, no substantial information exists on the ability of NK cells to kill autologous melanomas. An efficient NK-mediated lysis of autologous melanoma cells has been described in patients undergoing specific T cell therapy. This reflect the selection of melanoma variants characterized by the complete absence of surface HLA class I due to the loss of β_2_-microglobulin [18]. Only a single case of HLA class I^+^ melanoma cell line was analyzed for its susceptibility to lysis by autologous NK cells. However, these studies were neither conclusive nor sufficiently explanatory of the molecular mechanisms involved in lysis [19], [20].

In the present study we investigated, in an autologous setting the susceptibility to NK-mediated killing of melanomas cell lines isolated from 10 different patients. We could detect cases with different types of HLA class I alterations, thanks to a comparative evaluation of autologous normal cells. In addition, the role of different NK cell subsets and the involvement of their activating/inhibitory receptors in the recognition and killing of human autologous melanoma was comprehensively analyzed.

## Materials and Methods

### Ethics Statement

This study was approved by the Ethics Committee of the Istituto Nazionale Ricerca sul Cancro, Genoa, where patients underwent surgical resections of malignant melanoma lesions and received further therapeutic treatments and follow-ups. Patients gave written informed consent according to the Declaration of Helsinki.

### Derivation of the Primary Melanoma Cell Lines

We established ten primary melanoma cell lines from patients who underwent surgical resection of skin or lymph node metastases. Melanoma samples were obtained immediately after surgical resection at the Istituto Nazionale Ricerca sul Cancro (Genoa).

Briefly, fresh tumor samples were washed extensively with phosphate-buffered saline (PBS) and were processed by mincing the tissue with operative scissors. Mechanically dissociated tissues underwent enzymatic digestion with 150 U/mL hyaluronidase and 250 U/mL collagenase type IV (Sigma-Aldrich, St. Louis, Mo). The single-cell suspension was filtered through a 100-µm cell strainer, washed and then plated in RPMI 1640 (Lonza, Verviers, Belgium) medium supplemented with 10% FCS (BioWhittaker, Walkersville, MD). Effective melanoma growth was assessed using an anti-Melanoma (MCSP)-PE mAb (Miltenyi Biotech, Bergish Gladbach, Germany).

Primary melanoma cell lines were employed when all cultured cells were positive for the anti-melanoma mAb (usually around the third cell culture passage).

### Purification of Fresh NK Cell Lymphocytes and Generation of Polyclonal NK Cell Populations

Whenever available (six out of ten patients), peripheral blood lymphocytes were derived from patient's blood samples by centrifugation on a Ficoll-Hypaque gradient. For isolation of freshly NK cells, PBMCs were negatively selected using NK Isolation Kit (Miltenyi Biotech, Bergish Gladbach, Germany). For generation of polyclonal NK cell population, the highly purified magnetically selected NK cells were cultured on irradiated feeder cells in the presence of 100 U/ml recombinant interleukin 2 (IL-2) (Proleukin, Chiron Corp, Emeryville, CA) and 1.5 ng/ml phytohemagglutinin (PHA) (GIBCO BRL, Carlsbad, CA).

To obtain highly purified “HLA non-inhibited” NK cell subsets from bulk NK, cells were incubated with a mixture of mAbs reactive against KIRs recognizing HLA-I molecules of the patient and NKG2A. Negative cells were then FACS sorted directly using FACSAria (Becton Dickinson, Mountain View, CA) All sorted subsets used for the experiments always displayed purity above 97%.

### ^51^Chromium Release Assay

To analyze the ability of IL-2 activated, freshly isolated and sorted “HLA non-inhibited” NK cells to lyse autologous primary cell lines, NK cells were tested in 4-h ^51^Chromium release tests. Briefly, melanoma target cells were incubated with Na_2_^51^CrO_4_ for 1 hr at 37°C, exstensively washed and finally cultured with the different NK cell populations for 4 hours, either in the absence or in the presence of various mAbs. The supernatant from the culture was collected and the radioactivity counted and radioactivity measured on a gamma counter (Beckman, Milan, Italy). ‘Specific’ ^51^Cr release is calculated on the bases of the ratio{(sample release - spontaneous release)/(total release - spontaneous release)}. Assays were performed in triplicate at the indicated effector-target ratio. For masking experiments, mAb of IgM isotype were used: anti-NKp30 (F252 clone), anti-NKp44 (KS38 clone), anti-NKp46 (KL247 clone), anti-DNAM-1 (F5 clone) and anti-pan-HLA class (A6136 clone). Masking of NKG2D was performed with BAT221 mAb, of IgG1 isotype. All antibodies cited above were produced in our laboratory.

### CD107a Assay

To investigate the surface expression of the genetically detected HLA class I alleles and their potential inhibitory role on NK cell functions, a CD107a degranulation assay was performed. The autologous melanoma tumor cells were used as a target. To detect spontaneous degranulation, a control sample without target cells was included. An effector/target (E/T) ratio of 1.5∶1 (3.75×10^5^ effector cells: 2×10^5^ target cells in a volume of 200 µL) was used. An AlexaFluor647-conjugated anti-CD107a (eBioscence, San Diego, CA) was added in each well (5 µL/well) before incubation. Effectors and targets were then coincubated at 37°C for 4 hours either in the absence or in the presence of the anti-pan HLA class I mAb A6-136; after the first hour monensin (Sigma-Aldrich, St. Louis, MO), at a final concentration of 2 mM, was added to inhibit cell secretion, as previously described [21]. After 4 hours, cells were washed and stained with a mixture of mAbs reacting with KIRs and NKG2A to analyze the surface expression of CD107a on each single-inhibitory receptor positive NK cell subset.

### mAbs and Flow Cytometry Analysis

To characterize the surface expression of the activating receptor ligands on the tumor cell lines, a panel of mAbs was used: anti-MICA (clone BAM195), anti-Nectin-2 (clone L14) and anti-PVR (clone L95) produced in our laboratory. Anti-ULBP1 (clone M295), anti-ULBP2 (clone M310), anti-ULBP3 (clone M550) and anti-ULBP4 (clone M475) (all from Amgen Inc., Seattle, WT). The adhesion molecules CD54 and CD58 were detected with PE-conjugated anti-CD54 and anti-CD58 antibodies (Beckman Coulter, Fullerton, CA).

To evaluate surface expression of HLA-I molecules on tumor cells and autologous PHA blasts, anti HLA class I (clone W6/32) and anti-HLA-A2 (clone BB7.2) mAbs (both by ATCC, Manassas, VA), anti-HLA-A1,3,11,24 (clone 131 produced in our laboratory) and FITC-conjugated anti-HLA-Bw4 (One Lambda Inc., Canoga Park, CA) were used. Single-inhibitory receptor positive NK cell subsets were phenotypically isolated by the use of unlabeled mAbs anti-NKG2A (clone Z199), anti-KIR3DL/S1/KIR3DL2 (clone AZ158), anti-KIR2DL2/3/S2 (clone CH-L), anti-KIR3DL1/S1 (clone Z27) and anti-KIR3DL2 (clone Q66) (produced in our laboratory) and labeled mAbs PE- and FITC-conjugated anti-KIR2DL1/S1, PE-conjugated anti-KIR2DL2/3/S2(Miltenyi Biotech, Bergish Gladbach, Germany).

For indirect immunofluorescence staining, cells were stained with the primary mAb, followed by FITC- or PE-conjugated, isotype-specific, goat anti-mouse or goat anti-human second reagent (Southern Biotechnology Associated, Birmingham, AL). Negative controls included directly labeled and unlabeled isotype-matched irrelevant mAbs. For phenotypic analises, data were acquired on FACSCalibur (Becton Dickinson, Mountain View, CA). Data analysis was performed using CellQuest software (Becton Dickinson, Mountain View, CA).

### HLA Typing

To perform HLA class I serological typing, the microlymphocytotoxicity test was employed, according to the manufacturer's instruction. Lymphocytes, obtained after ACD-treated peripheral blood centrifugation, were resuspended in Hank's balanced salt solution without Ca and Mg (Biotest AG, Dreieich, Germany) and magnetic microbeads coated with anti-CD8 monoclonal antibody (One Lambda Inc., Canoga Park, CA) were added. Cell-linked microbeads were placed in a magnetic separator (Biotest AG, Dreieich, Germany) and the T cell suspension obtained, was dispensed to each well of Terasaki HLA class I 144 microplates (Lagitre S.R.L, Milan, Italy).

To carry out genomic typing, DNA was extracted using the QIAamp DNA blood mini kit (QIAGEN GmbH, Hilden, Germany), according to the manufacturer's instruction. To identify HLA alleles, it was employed the PCR – SSO Reverse assay using HLA Kits (Innogenetics N.V., Gent, Belgium). The assay was performed according to the manufacturer's instruction. Data analysis was performed using the LIPA Interpretation Software (Innogenetics N. V., Gent, Belgium).

To evaluate HLA-E mRNA levels on primary melanoma cell lines total RNA was extracted using RNeasy micro kit (QIAGEN GmbH, Hilden, Germany) according to the manufacturer's instruction. The cDNA synthesis was performed on about 1000 ng of RNA using oligo-dT oligonucleotides. To selectively amplify the second and the third exons of HLA-E we used the set of primers HLA-E up 5′ ACA CCG CAC AGA TTT TCC and HLA-E down 5′ AGG GTG GCC TCA TGG TC. PCR were performed for 30 cycles: 30″ at 95°C; 30″ at 56°C; and 30″ at 72°C.

### Statistical Analysis

Statistical analyses were performed using the Graph- Pad Prism v. 4.00 for Windows (GraphPad Software, San Diego, Calif). Statistical significance was evaluated by paired Student t-test.

## Results and Discussion

### IL-2-Activated NK Cells Effectively Kill Autologous Melanoma Cells

**Figure 1:**
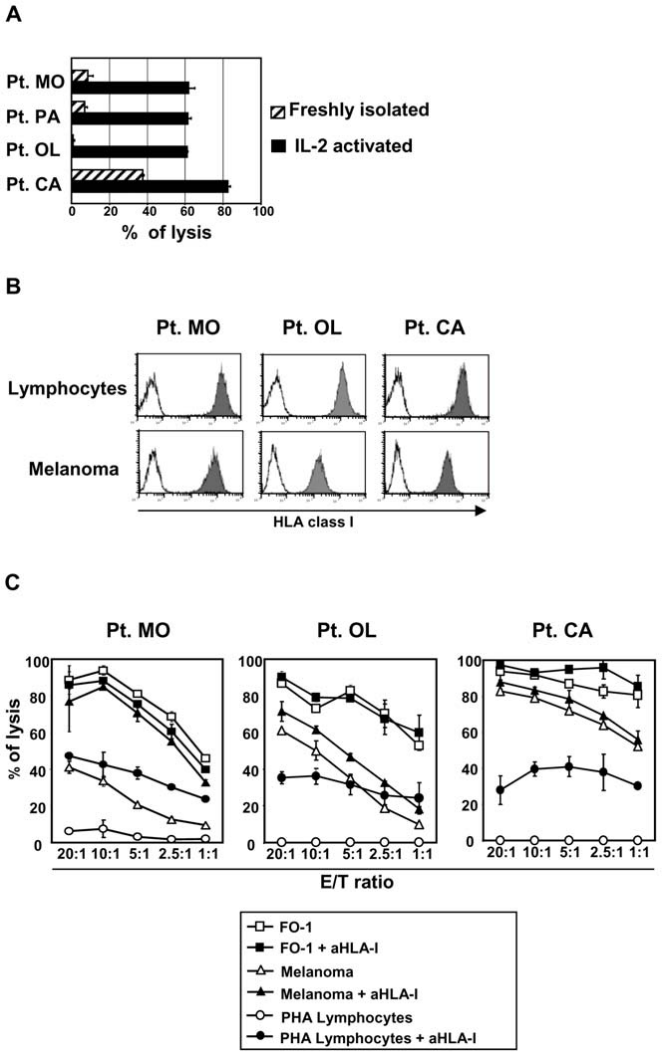
Cytolitic capabilities of human NK cells against autologous melanoma. **A:** Susceptibility of melanoma cells to autologous NK cell-mediated lysis. Freshly isolated or IL-2 activated NK cells were used as effector cells in a specific ^51^chromium release assay. The effector/target (E/T) ratio used was 20∶1. Four representative patients are shown and results are expressed as means±SD derived from experiments in triplicate. **B:** Flow cytometry evaluation of total HLA class I expression (W6/32 mAb) on melanoma cells and, as a comparison, on autologous lymphocytes. **C:** Susceptibility to NK-mediated lysis of melanoma cells compared with susceptibility of HLA class I-deficient FO-1 cell line and autologous lymphocytes stimulated with PHA (PHA lymphocytes). Cytolitic activity was assessed by ^51^chromium release tests. In order to evaluate the inhibitory effect caused by melanoma HLA class I expression on NK cell activity, lysis of FO-1, autologous melanoma cells and PHA lymphocytes was also analysed in the presence of the anti-pan HLA class I blocking mAb (A6-136, IgM) at the indicated E/T ratios. At least three experiments were performed for each patients. Representative experiments of three patients out of six are shown. Data represent mean value±SD of experiments performed in triplicate.

We first assessed the ability of NK cells to recognize and kill autologous primary melanoma cell lines by comparatively analyzing the cytotoxic capabilities of freshly isolated or IL-2 activated polyclonal NK cells. These experiments (using a specific chromium release assay) showed that NK cells require cytokine activation for an efficient killing against autologous melanomas while, in most cases, the lytic capabilities of unactivated NK cells were limited (Fig. 1A).

Because autologous NK cells displayed high levels of cytolytic activity when activated, we further investigated the level of expression of HLA class I molecules on 10 established primary melanoma cell lines, and, whenever possible, as a comparison, on PHA-activated lymphocytes obtained from the same patients. We found that HLA class I was always well expressed at the melanoma cell surface, in some istances in amounts comparable to autologous lymphocytes. The analyses of three representative patients out of ten investigated are shown in figure 1B.

Next, the susceptibility of primary melanoma cell lines to NK cell mediated lysis was compared to that of the HLA class I negative melanoma cell line FO-1 or PHA-activated autologous lymphocytes (Fig. 1C). In all cases, melanoma cells were efficiently lysed by autologous NK cells even at levels similar to FO-1 cells. On the contrary, autologous PHA blasts, were always resistant to lysis. Pretreatment of target cells with saturating amounts of anti-pan HLA class I mAb resulted in an increase in lysis of PHA-blasts, while variable degrees of increases were detected in different melanoma cell lines. As expected, addition of the anti-HLA class I blocking mAb had no effects on the lysis of HLA-class I-negative FO-1 cells. Remarkably, in certain melanomas, no increase in their susceptibility to lysis was detected upon HLA class I masking despite their high expression of (total) HLA class I on their surface.

Taken together, these results suggest that the melanoma cells analyzed might display defects in the expression of HLA class I alleles interacting with inhibitory KIR expressed by autologous NK cells.

### Melanoma Cells May Display Deletions of HLA Class I Alleles that Interact with KIR Expressed by Autologous NK Cells

Since melanoma cells were susceptible to NK-mediated lysis despite the high levels of (total) HLA class I expression, we investigated whether there were deletions of HLA class I locus/alleles specifically recognized by autologous NK cells. Notably, as previously reported^12^, selective loss of HLA class I alleles occurs frequently in melanoma. To this end HLA class I typing was performed in melanoma cells in comparison with autologous peripheral blood lymphocytes. HLA class I alleles (A, B, C) were assessed by applying both DNA-based methods and serological typing. We also evaluated by RT-PCR the HLA-E mRNA expression in each melanoma cell line.

These analyses revealed allele-specific deletions in half of the primary melanoma cell lines studied. In addition, most deletions affected alleles specifically interacting with KIRs expressed by autologous NK cells. We found deletions at different level: some alleles were deleted at genomic level as the cells lacked the gene coding for the specific allele product, whereas other alleles were present at genetic level but not detected on the cell surface. The various defects in HLA class I allele expression detected in primary melanoma cell cultures are summarized in Table 1.

**Table 1:** Comparative HLA class I typing in normal lymphocytes and melanoma cells.

	HLA-A	HLA-A	HLA-B group	HLA-B group	HLA-C	HLA-C
	*Lymphocytes*	*Melanoma*	*Lymphocytes*	*Melanoma*	*Lymphocytes*	*Melanoma*
**Pt. MO**	A2/A29	A2/A29	**Bw4**/Bw6	**Bw4**/Bw6	**Cw6**/**Cw16**	**Cw6/Cw16**
**Pt. CO**	A2/**A3**	XX/**A3**	**Bw4**/Bw6	**XX**/XX	**Cw4/Cw5**	**XX/Cw5**
**Pt. PA**	A26/A32	A26/XX	**Bw4**/Bw6	**Bw4**/X	**Cw2/Cw4**	**XX/Cw4**
**Pt. MT**	A1/A25	A1/A25	Bw6/Bw6	Bw6/Bw6	**Cw4/Cw7**	**Cw4/Cw7**
**Pt. OL**	A26/A30	A26/A30	**Bw4**/Bw6	**Bw4**/Bw6	**Cw6/Cw8**	**Cw6/Cw8**
**Pt. CA**	A1/A1	A1/A1	**Bw4**/Bw6	**X**/Bw6	**Cw4/Cw7**	**Cw4/X**

Comprehensive HLA class I typing for each patient are shown. HLA-A,-B and -C alleles relevant for the NK cell inhibition are in bold. (X) indicates that the gene coding for the antigen was present but the HLA protein was not expressed on the cell surface (detected by serologic typing – Terasaki method and, when possible according to antibody availability, also by flow cytometry), whereas (XX) indicates that the gene coding for the antigen was deleted.

**Figure 2:**
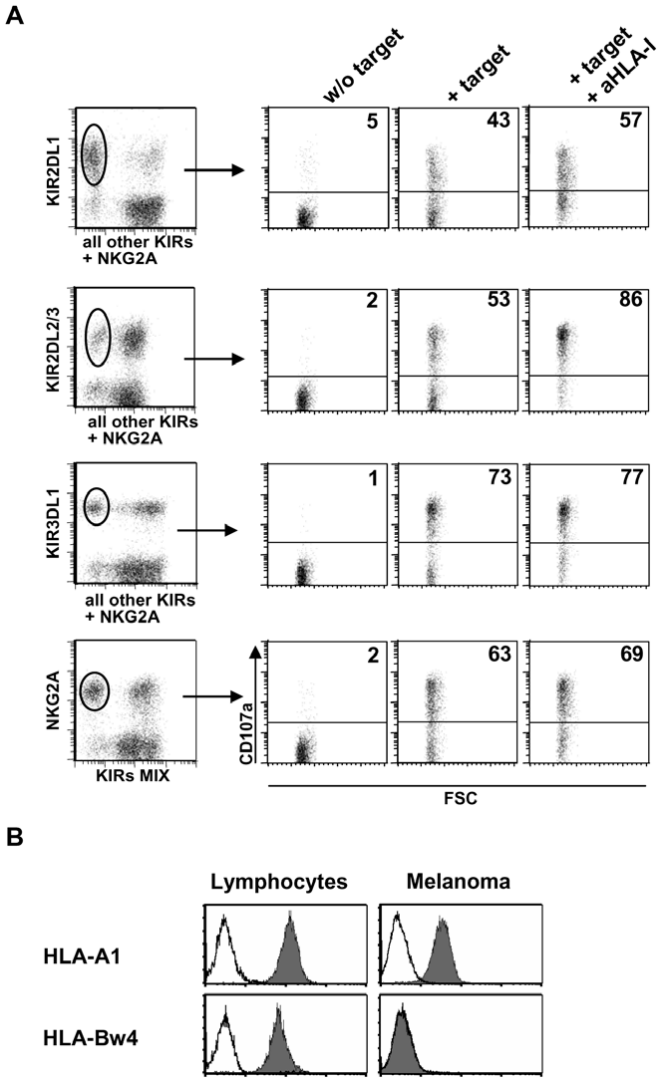
CD107a degranulating assay on distinct NK cell subsets expressing single inhibitory receptors. CD107a degranulating ability of activated NK cell subsets from each patient was analyzed in the presence of autologous melanoma cells, either adding or not an anti-pan HLA class I blocking mAb. E/T ratio = 1.5∶1. A representative analysis of patient CA is shown in panel **A**. In order to assess the link between HLA class I expression on melanoma cells and their susceptibility to autologous NK cell-mediated lysis, the degranulation of NK cell subsets, expressing a single inhibitory receptor for distinct HLA class I molecules, was analyzed. Staining NK cells with mAbs against a specific KIR or NKG2A and with a mixture of mAbs against the other inhibitory receptors allowed to gate on the specific subset and evaluate its CD107a expression after co-culture with autologous melanoma cells. **B**: Melanoma specific deletion of a HLA class I allele in the same patient CA. HLA-Bw4 was here detected by flow cytometry on the surface of lymphocytes but not of the autologous melanoma. As a comparison, another conserved HLA-A allele is shown. In accordance with Bw4 deletion, KIR3DL1+ NK cell subset exerts a strong cytolytic activity against autologous melanoma cells, which is not significantly increased following anti-HLA class I masking.

**Figure 3:**
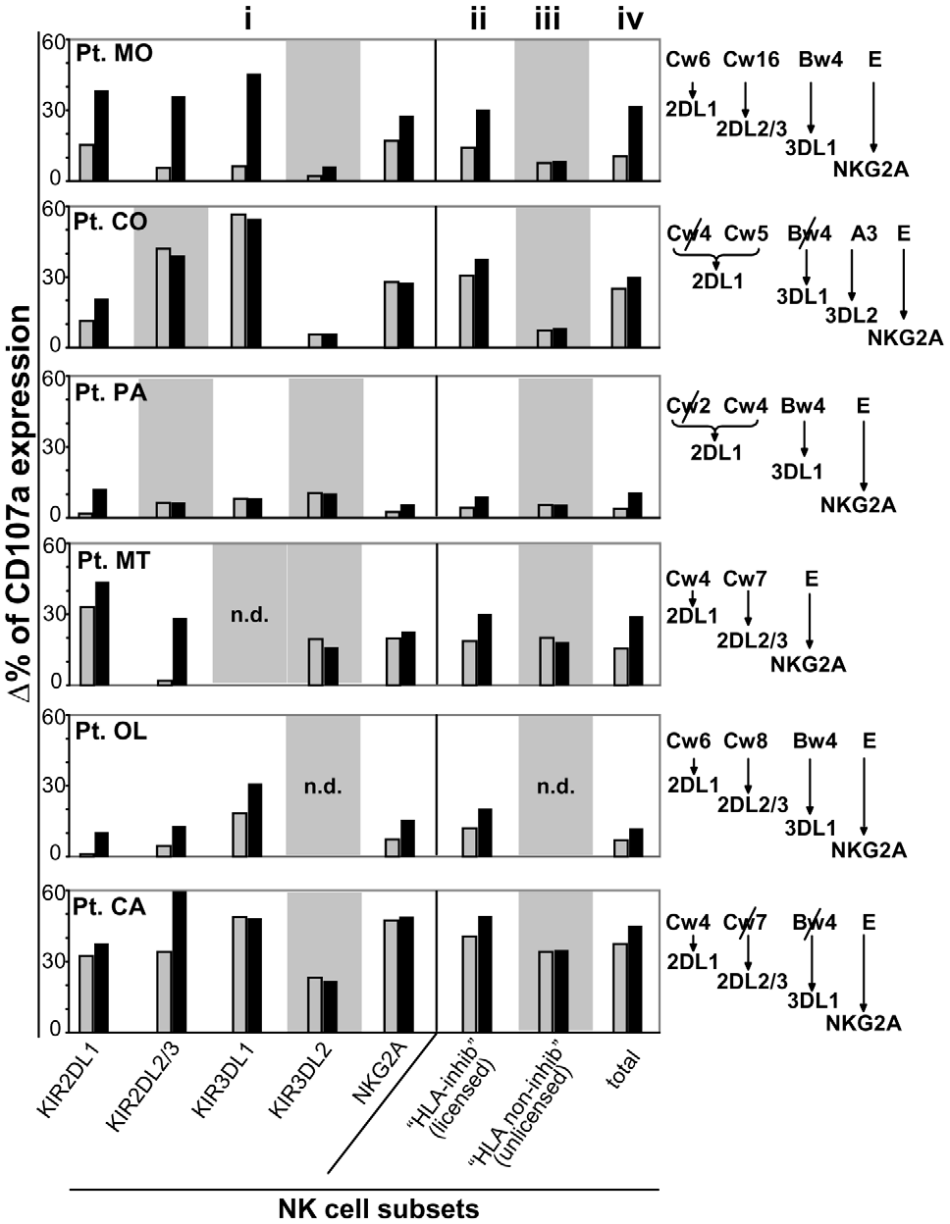
Role of HLA class I alteration in melanoma susceptibility to lysis by autologous NK cells. Summary of CD107a degranulating assays (performed as in figure 2A) analyzed on NK cell subsets in the presence of autologous melanoma cells from six patients. Degranulation was analyzed on (i) NK cell subsets expressing the single inhibitory receptors, (ii) NK cell subsets expressing one or more inhibitory receptors (“Total HLA-inhibited”), (iii) NK cell subsets expressing no inhibitory receptors for self HLA (“Total HLA non-inhibited”) and (iv) the whole NK cell population. Data represent percentages of CD107a expression in the presence of autologous target (grey columns) or in the presence of autologous target plus anti-HLA class I mAb (black columns). Values were calculated by subtracting the spontaneous CD107a expression in the absence of the target. NK cell subsets expressing no receptors for self-HLA are depicted on a grey background. n.d. indicates that the specific NK cell subset was not detectable in a given patient. For each patient, on the right side of the figure, patient HLA class I antigens that are relevant for inhibiting NK cell activity are shown and antigens that have undergone deletions on autologous melanoma cells are marked by a slash. For HLA class I A,B,C, both molecular and serological typing were performed (see also Table 1), while HLA-E was detected only at mRNA level. For each HLA class I antigen, matched inhibitory receptor is also specified. Results are expressed as means of 3 to 6 independent experiments. E/T ratio was 1.5∶1.

Deletions of specific HLA class I alleles may account for the susceptibility to NK cell mediated lysis described above. In order to directly assess this possibility, CD107 degranulation assay was performed in NK cells co-cultured with autologous melanoma cells. In particular, degranulation was evaluated in NK cells expressing single receptors specific for alleles that were either expressed or deleted on melanoma cells. As shown in figure 2 in a representative patient (patient CA of table 1) and described in more details in the Material and Methods section, surface CD107a was analyzed in NK cell subsets expressing a single receptor. Experiments were performed (by multicolor flow cytometry) in polyclonal NK cells after co-culture with autologous melanoma cells, either in the presence or in the absence of anti-pan HLA class I mAb. The degrees of degranulation were calculated by subtracting the spontaneous degranulation occurring in the absence of the melanoma target. Mean degranulation values detected in each NK cell subset of six patients, upon co-culture with autologous melanoma cells, are shown in figure 3.

In line with the data of HLA typing, NK cells expressing a single inhibitory receptor specific for a deleted allele displayed an efficient degranulation upon co-culture with autologus melanoma cells (e.g. patient CO and patient CA KIR3DL1; figure 3). Addition of anti-pan HLA class I mAb (A6-136, IgM) did not result in any increase of cytolytic activity. In some istances, NK cells expressing KIR specific for a deleted allele interacted with another allele belonging to the same HLA-C specificity (e.g. in KIR2DL1 patients CO and PA). With regard to the NKG2A inhibitory receptor, HLA-E mRNA was detectable in all melanoma cell lines analyzed (data not shown). However, addition of anti-pan HLA class I blocking mAb not always increased the degranulation of NKG2A^+^ NK cell subsets during co-culture with the related autologous melanoma cells (Figure 3). In particular, in patients CO and CA the surface expression of HLA-E appears virtually absent while at least decreased in patient MT. These findings might suggest that, in agreement with previous reports [22], [23], HLA-E is often scarcely represented on melanoma cell surface.

In contrast, in most of the NK cells expressing a single receptors for alleles conserved in autologous melanoma cells, addition of anti-pan HLA class I blocking mAb resulted in increases of degranulation (Figure 3).

### Expression of Low Amounts of HLA Class I Molecules May Contribute to the NK Cell-Mediated Killing of Melanoma Cells

Table 1 shows that, in some instances, the melanoma cells analyzed expressed HLA class I alleles recognized by inhibitory KIR, as revelead by specific mAbs and serological HLA typing. Accordingly, NK cells expressing a single KIR specific for alleles expressed by melanoma cells generally displayed low degree of degranulation upon co-culture with autologous melanoma cells (Figure 3). In these instances, degranulation increased in the presence of anti-HLA class I blocking mAb. Nevertheless, in some cases, as shown in figure 3, also NK cells expressing single inhibitory KIR underwent degranulation despite the expression of cognate HLA ligand on autologous melanoma cells. In patient MT, for instance, Cw4 (i.e. the ligand of KIR2DL1) was expressed but KIR2DL1+ NK cells could effectively degranulate, strongly suggesting that Cw4-mediated inhibition was partially uneffective. Addition of anti-pan HLA class I further increased degranulation in such KIR2DL1+ NK cells. Similar results were obtained in patient OL (for KIR3DL1+ NK cells; ligand Bw4) and in patient CA (KIR2DL1+ NK cells; ligand Cw4). A possible explanation for these findings might be a variable expression of HLA alleles in melanoma cells of our primary cell lines. However, analysis of several melanoma cell clones obtained by limiting dilutions led to similar results (not shown). Therefore, in agreement with previous data [19], NK-mediated killing may also reflect a low expression (and not the absence) of a given HLA-allele, that is not sufficient to deliver a suitable inhibitory signal to NK cells.

A particular case was represented by patient CA. In this case the inhibitory HLA ligand for KIR2DL2/3+ NK cells was lacking on autologous melanoma target cells. However, some increase of degranulation was detected upon HLA class I masking. A likely explanation for this result is suggested by a recent report showing that Cw4 (conserved on this patient melanoma cells) can bind with low affinity KIR2DL2/3 [24].

Taken together, these data showed that: 1) HLA-E is frequently absent on melanoma cell surface, accordingly, degranulation of NKG2A+ NK cells was, in most cases, virtually unchanged in the presence of anti-pan HLA I blocking mAb; 2) in the presence of deletions of specific HLA class I alleles melanoma cells are susceptible to lysis by autologous NK cells; 3) although conserved in melanoma cell surface, specific HLA class I alleles may be expressed in amounts insufficient to inhibit NK cells.

### “Unlicensed” NK Cells Are Efficient in Killing Autologous Melanoma

It has recently been shown that NK cells lacking inhibitory receptors for self MHC class I molecules are well represented in human peripheral blood [17]. Such “HLA-non-inhibited” NK cells, display a mature NK cell phenotype but are hyporesponsive to various stimuli, including interaction with HLA class I-deficient target cells. Indeed, because of this functional characteristics, they have been previously termed “unlicensed” NK cells [16], [17]. However, their possible activity against HLA-class I+ autologous tumor cells expressing HLA class I is unknown. Notably, it has been reported that they may improve their functional potential upon cytokine activation [16]. Thus, we analyzed whether “HLA non-inhibited” NK cells could contribute to killing of autologous melanoma cells.

**Figure 4:**
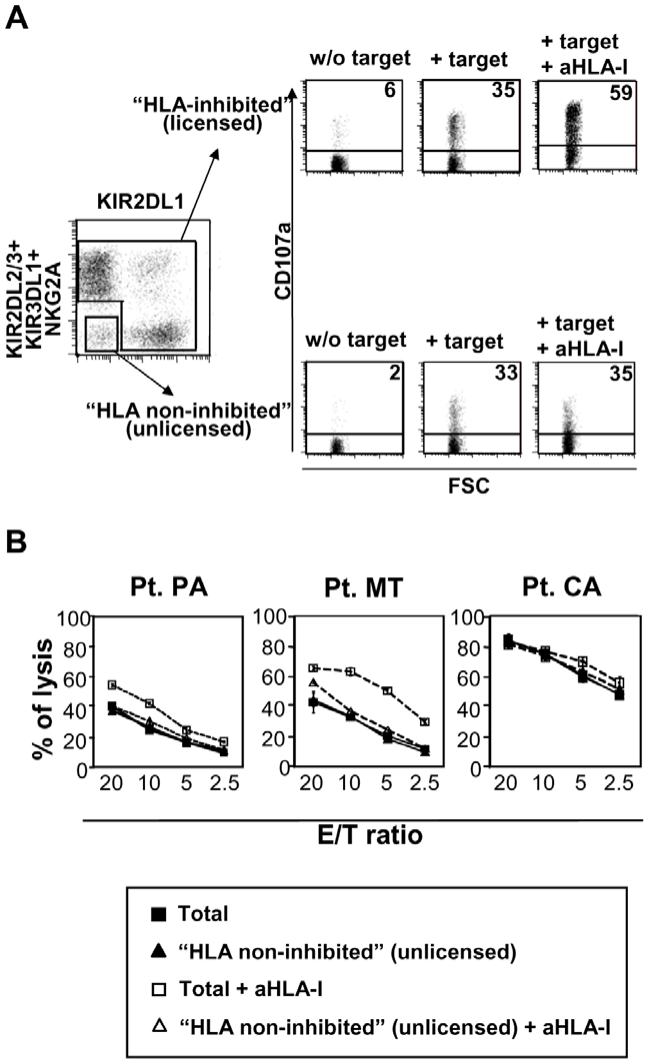
Cytolytic activity against autologous melanoma of NK cell subsets expressing no inhibitory receptors for self-HLA class I. The cytolytic activity of NK cell subsets expressing no inhibitory receptors for self-HLA class I (“HLA non-inhibited”) was compared with the counterpart expressing at least one receptor for self-HLA. **A:** Cytolytic activity was assessed by CD107a degranulation assay for total “HLA-inhibited” and total “HLA non-inhibited” NK cell subsets. A representative experiment is shown. Briefly, triple fluorescence staining with a mixture of PE-conjugated anti-KIR2DL1 mAb and a mixture of other anti-KIR (KIR2DL2/3 and KIR3DL1) and anti-NKG2A mAbs allowed to gate on the KIR^−^/NKG2A^−^ subset plus NK cell subset expressing receptors for HLA class I alleles not expressed by the related patient. On the other hand, the staining permitted also to gate on all “HLA-inhibited” subsets. Thus, as shown in panel A, CD107a expression of the whole “HLA non-inhibited” subset was analyzed and compared with that of whole “HLA-inhibited” subset, in the presence of absence of anti-HLA class I blocking mAb. In agreement with the absence of inhibitory receptors for self HLA, “HLA non-inhibited” NK cell subset did not increase their cytotyitc activity in the presence of anti-HLA class I masking mAb. A summary of all experiments performed in each patients is shown in figure 3B(ii,iii,iv) . **B:** The effectiveness of “HLA non-inhibited” NK cell subsets in killing autologous melanoma was also assessed by a standard ^51^chromium release assay. The whole “HLA non-inhibited” NK cell populations derived by three representative patients were sorted out by flow cytometry as double negative cells and compared with the unstained total bulk NK populations. The comparison was performed with the unstained total NK cells, rather than with HLA-inhibited NK cell subets, to avoid possible interference caused by antibody binding to functional receptors. Results shown are from three patients and data correspond to mean values±SD of experiments performed in triplicate.

The “HLA non-inhibited” NK cell fraction is composed of NK cells lacking both inhibitory KIR and NKG2A and of NK cells that express only inhibitory KIR that do not interact with self HLA class I molecules. A representative experiment, using a multicolour flow cytometric assay is shown in figure 4A (patient CA). Confirming their lack of inhibitory receptors for self HLA class I molecules, “HLA non-inhibited” NK cells did not increase their degranulation in the presence of anti-pan HLA class I blocking mAb (Figure 4A). Surprisingly, analysis of the “HLA non-inhibited” NK cell subset did not show major differences in CD107a expression as compared to conventional (“HLA-inhibited”) NK cells when tested against autologous melanoma cells. Indeed, levels of degranulation were similar in four out of six patients. Only in one case (Patient CO) CD107a expression was lower in “HLA non-inhibited” NK cells (Figure 3 ii, iii, iv).

The cytotoxic activity of “HLA non-inhibited” NK cell subsets was also tested in a ^51^chromium release assay. “HLA non-inhibited” NK cells were separated by sorting from “HLA-inhibited” ones (as cells that were not stained by mAbs specific for the various inhibitory receptors) ruling out possible artifacts caused by bound antibodies. Therefore, “HLA non-inhibited” cells were compared to unfractioned, unstained NK cells. As shown in figure 4B, the three autologous melanoma cell lines tested were lysed to the same extent by “HLA non-inhibited” NK cells and unfractioned NK cells (largely represented by conventional “HLA-inhibited” NK cells). Again, in contrast to “HLA inhibited” NK cells, the cytotoxic activity of “HLA non-inhibited” was not increased by the addition of anti-pan HLA class I blocking mAb (Figure 4B).

Thus, although “HLA non-inhibited” cells had been reported to be poorly effective in killing HLA-I negative target cells, upon IL-2 activation, they acquire both relevant degranulating capacity and cytotoxic activity against autologous melanoma cells. Since this NK cell subset does not express inhibitory receptors recognizing patient HLA-class I, it is conceivable that their lower cytototoxic potential may be compensated by the complete absence of inhibitory signals delivered via HLA molecules. In conclusion, “HLA non-inhibited” (unlicensed) NK cells, if properly activated, can effectively recognize and kill autologous tumor cells.

### NK Cells Recognize and Kill Autologous Melanoma Cells Primarly via DNAM-1 and NKp46 Activating Receptors

**Figure 5:**
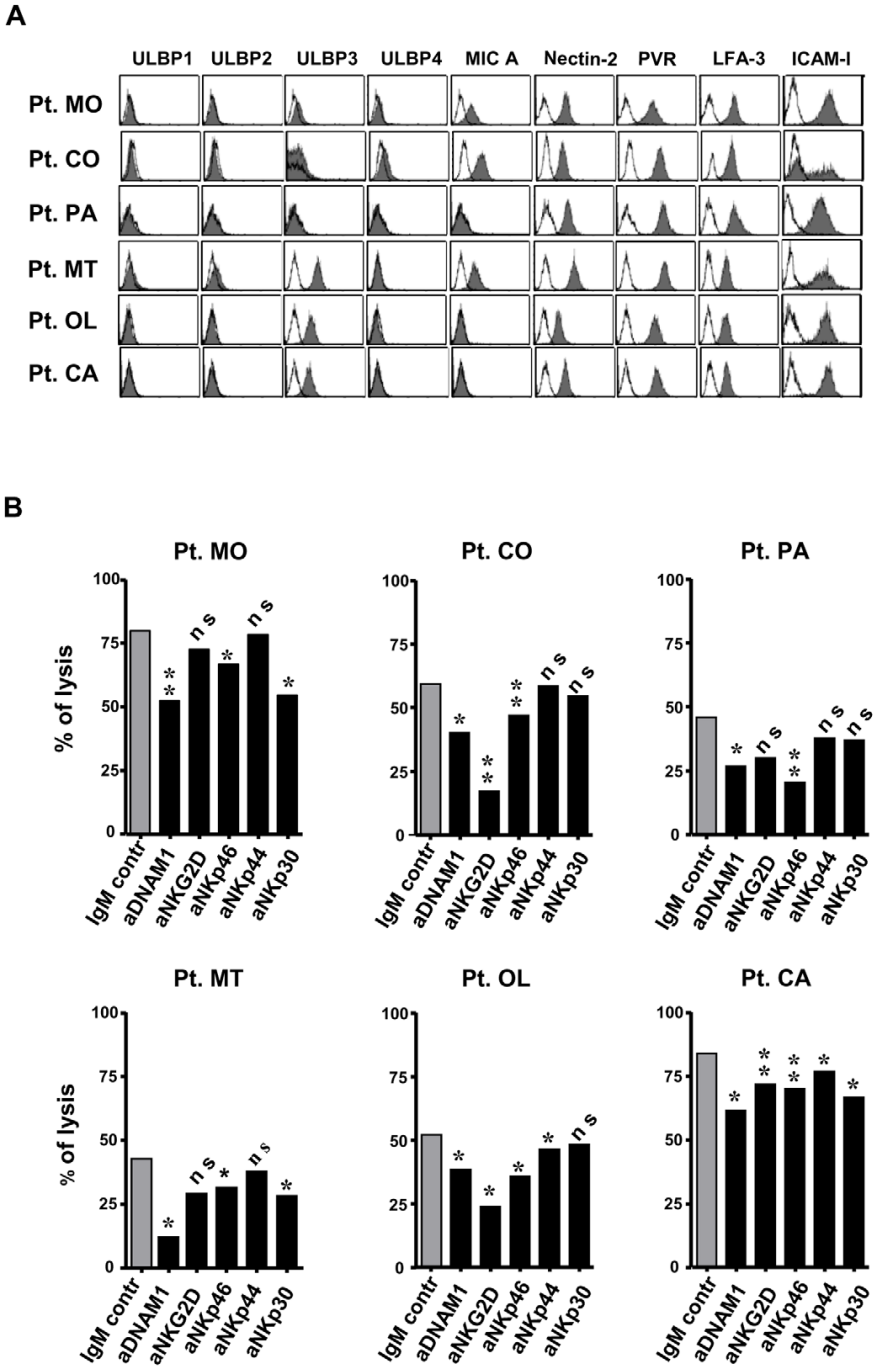
Melanoma surface molecules and NK cell activating receptors involved in the lysis of melanoma cells by autologous NK cells. **A:** Melanoma cell expression of molecules able to trigger NK cell activity. Representative data are shown for the six melanoma cell lines whose autologous lymphocytes were also available for performing the functional experiments summarized in panel B. Melanoma cells were stained with mAbs against ligands of NKG2D and DNAM-1 and with mAbs against adhesion molecules LFA-3 and ICAM-I. **B:** Contribution to the lysis by different activating NK cell receptors. Cytotoxic activity of IL-2 activated NK cells from each patient against the autologous melanoma cells was assessed in the presence of anti-DNAM-1, -NKp30, -NKp44, -NKp46 and -NKG2D (black columns) and isotypic control (grey column) mAbs. *: p≤0.05; **: p≤0.01; n.s.: not significant. E/T ratio = 20∶1. Data shown represent mean values of the results obtained from three to six independent experiments.

Further experiments were designed to analyze in ten primary melanoma cell lines the pattern of expression of the known DNAM-1 and NKG2D ligands. Cytofluorimetric analysis revealed that the DNAM-1 ligands PVR and Nectin-2 were expressed by all cell lines (PVR at a higher level than Nectin-2). Conversely, the NKG2D ligands displayed a variable pattern of expression. Thus, MICA was detected in five melanoma cell lines while among ULBPs, only ULBP-3 and ULBP-4 were detectable on four and one melanoma cell lines, respectively. Only a single melanoma cell line was negative for all NKG2D ligands. Finally, all tumor cell lines expressed the adhesion molecules ICAM-1 and LFA-3 with the exception of a single melanoma cell line (patient CO) which expressed ICAM-1 in a fraction of the cells only. Figure 5A shows the expression pattern of these molecules in six representative melanoma cell lines.

In parallel with the phenotypic analysis, we also evaluated the contribution of each activating NK receptor to autologous melanoma cell lysis. In these experiments, the NK cell cytotoxicity was analyzed by the ^51^chromium release assay in the presence or absence of blocking mAbs specific for DNAM-1, NKG2D or NCRs to prevent the interaction between receptors and their specific ligands on target cells. Results from six cell lines are shown in figure 5B. In agreement with the finding that PVR and Nectin-2 were expressed by all cell lines, in all instances an efficient target cell lysis was detected. In addition, this was significantly reduced in the presence of anti-DNAM-1 mAbs, indicating that DNAM-1 plays a relevant role in NK-mediated lysis of melanomas. Although to a lesser extent, also NKp46 receptor appeared involved in the lysis, since, similar to DNAM-1, blocking this receptor consistently reduced the lysis of all melanomas analyzed. Conversely, NKp30 receptor was involved in the lysis of three patients out of six. In addition, despite MICA and ULPBs were detectable on five melanoma cell lines, only in three cases a statistically significant reduction of the lysis could be detected upon NKG2D blocking. However, anti-NKG2D blocking mAb consistently synergized with either anti-DNAM-1 or anti-NCRs mAb in the inhibition of NK cell cytotoxicity (not shown). Finally, blocking of NKp44 resulted in limited decrease of cytotoxicity. Collectively, these results suggested that the susceptibility of melanomas to NK cell-mediated lysis is due to the expression of a variety of ligands for activating NK receptors, in particular PVR and Nectin-2, the DNAM-1 ligands. Although different NKG2D ligands could be detected in some of the melanoma cell lines, this receptor appears only partially involved in melanoma cell killing, as recently shown also in other experimental models of melanoma [25], [26].

### Concluding Remarks

The molecular programme regulating NK cell activation and killing is a complex system composed of diverse inhibitory receptors, sensing primarly MHC-class I expression and of activating receptors interacting with specific ligands on target cells. In this study, by establishing primary melanoma cell lines from different patients, we showed that melanoma cells can be effectively killed by autologous NK cells. The mechanisms responsible for the lysis have been analyzed in detail. We found that the activanting receptors preferentially involved in killing were DNAM-1 and NKp46. In some instances, killing was consequent to specific losses on melanoma cells of HLA-I alleles recognized by KIR expressed by autologous NK cells. In other cases, there was a low expression of these alleles, not sufficient to deliver effective inhibitory signals. Remarkably, we highlighted a novel mechanism of NK cell-mediated lysis that is independent of HLA class I expression by melanoma. Thus, tumor cell lysis could be mediated also by the so called “unlicensed” NK cells, i.e. NK cells expressing no inhibitory receptors for self HLA class I. These NK cells are present in significant proportions in peripheral blood of patients as well as of normal donors and may therefore play a relevant role in killing autologous melanomas upon appropriate activation. Our present data may suggest a more general role of this NK cell subset in limiting and counteracting the spread of tumor cells in vivo, particularly under conditions in which they are activated (e.g. by IL-2). Notably, in spite of the lack of HLA-I-specific I inhibitory receptors, their cytolytic activity is mostly directed against tumor or otherwise stressed cells. This reflects the expression on these cells, of the ligands of activating NK receptors, absent or expressed in low amounts in normal cells. That this may indeed be the case is revealed by the lack of NK-mediated GvHD in haploidentical HSC transplantation in which “alloreactive” (i.e. KIR-HLA-class I-mismatched) NK cells play a major role against leukemic cells but do not attack normal allogeneic cells of the recipient [27].

In conclusion, activated human NK cells are highly effective, at least *in vitro*, against autologous melanoma cells via mechanisms that may be related or not to abnormalities of HLA class I expression in melanoma cells. However, in order to successfully utilize NK cells in the design of novel cancer immunotherapy approaches, it still remains mandatory to improve our knowledge regarding the trafficking of the various NK cell subsets towards neoplastic lesions as well as analyzing their functional properties (and effectiveness) within human malignancies.
